# *Nosema ceranae*, Fipronil and their combination compromise honey bee reproduction via changes in male physiology

**DOI:** 10.1038/s41598-017-08380-5

**Published:** 2017-08-17

**Authors:** Guillaume Kairo, David G. Biron, Faten Ben Abdelkader, Marc Bonnet, Sylvie Tchamitchian, Marianne Cousin, Claudia Dussaubat, Boris Benoit, André Kretzschmar, Luc P. Belzunces, Jean-Luc Brunet

**Affiliations:** 1 INRA, UR 406 Abeilles & Environnement, Toxicologie Environnementale, CS 40509, 84914 Avignon Cedex 9, France; 2 CNRS, UMR CNRS 6023 Laboratoire Microorganismes: Génome et Environnement, 63177 Aubière Cedex, France; 3 INAT, Laboratoire de Zoologie et d’Apiculture, 1082 Tunis, Tunisia; 4 INRA, UR 546 Biostatistiques & Processus Spatiaux, CS 40509, 84914 Avignon Cedex 9, France

## Abstract

The honey bee is threatened by biological agents and pesticides that can act in combination to induce synergistic effects on its physiology and lifespan. The synergistic effects of a parasite/pesticide combination have been demonstrated on workers and queens, but no studies have been performed on drones despite their essential contribution to colony sustainability by providing semen diversity and quality. The effects of the *Nosema ceranae*/fipronil combination on the life traits and physiology of mature drones were examined following exposure under semi-field conditions. The results showed that the microsporidia alone induced moderate and localized effects in the midgut, whereas fipronil alone induced moderate and generalized effects. The parasite/insecticide combination drastically affected both physiology and survival, exhibiting an important and significant generalized action that could jeopardize mating success. In terms of fertility, semen was strongly impacted regardless of stressor, suggesting that drone reproductive functions are very sensitive to stress factors. These findings suggest that drone health and fertility impairment might contribute to poorly mated queens, leading to the storage of poor quality semen and poor spermathecae diversity. Thus, the queens failures observed in recent years might result from the continuous exposure of drones to multiple environmental stressors.

## Introduction

Through reproduction, sexual species have the ability to respond to environmental stressors in their habitats by producing adapted offspring. However, pollutants and biological agents might threaten sexual reproduction by impairing fertility. For instance, in humans, infectious agents such as fungi, bacteria and viruses^1–3^ as well as xenobiotics such as endocrine disruptors and pesticides cause fertility impairment in individuals of both sexes^4–6^. Similar impairments have been observed more broadly in wildlife, including vertebrates and invertebrates^7–9^. In insects, reproductive disorders induced by environmental stressors can result in fertility decline^10–21^, a change in reproductive behavior, such as choice of sexual partner(s) and mating success^22, 23^, and diverse effects on offspring production and physiology^10, 24–29^.

The effects of numerous stressors on the health of organisms have gained increasing interest because of possible impacts not only on animal health but also on ecosystem services^30, 31^. For instance, potential drivers of pollinator loss (ex.: pesticides, pathogens, and the interactions between them) could result in weakening of pollination services in ecosystems, resulting in significant negative ecological (ex.: wild plant diversity) and economic (ex.: crop production) impacts^32, 33^. Among various stressors, the combined effects of pollutants and simultaneous infection by parasites on host health are of primary concern^30, 31^. Depending on the stressor combination, the joint effects can be antagonistic, additive, synergistic or potentiating. A synergistic interaction is defined as a combination of stressors that results in a greater effect than the cumulative expected effect from independent exposure^34^. The synergistic interaction of chemicals combined with natural stressors, such as pathogens, has been studied in aquatic organisms, such as *Daphnia* and *Artemia* ^30, 35^, and, more recently, in the honey bee^36–43^. Co-exposure might induce additive or synergistic effects, as shown in honey bee workers^36–39, 41^ and queens^42^ following exposure to *Nosema Ceranae* microsporidia and several insecticides. However, the drone caste has not been examined despite evidence that reproductive disorders can lead to species weakening. These disorders have rarely been suggested to explain the pollinator decline that has been observed worldwide, particularly in the decline of bee populations exposed to stressors^32, 44^. The honey bee, *Apis mellifera* (Hymenoptera: *Apidae*), is a valuable host model to study the ecological and evolutionary dynamics of host/parasite/pesticide interactions in eusocial insects because of its economic value, genetic diversity, available data on its biology and parasites, and pioneer studies on honey bee/parasite/pesticide interactions.

The honey bee, a species reported to be declining in the Northern hemisphere^45, 46^, has a singular mode of reproduction that uses a haplodiploid sex-determination system and polyandry. Thus, at an early stage of its life, a young diploid queen mates with up to 20 haploid drones in specific sites called drone congregation areas, where fierce competition occurs due to a highly male-biased sex ratio^47, 48^. Following mating, a small proportion of the spermatozoa received from each drone migrates into spermatheca to be used by the queen to fertilize eggs throughout her life^47, 48^. The diversity of sperm stored, a result of the polyandry, enables high intra-colonial genetic diversity that enhances survival, fitness, productivity and resilience to environmental disturbances^49–51^. Moreover, sperm diversity plays an important role, but sperm quality is also extremely important because drone semen quality determines spermathecal content, which is closely linked to queen reproductive potential^20^. Thus, a drone is a key individual at the origin of a cascade of events required for colony growth and sustainability. However, although drones are key to colony health, the effects of drone exposure to environmental stressors have been poorly investigated. Some studies, mostly focused on the effects of *Varroa* mites and mite treatments, have shown that drone survival^52–54^, fertility^11, 12, 55^ and mating behavior^12^ are impacted. More recently, it has been shown that other environmental stressors, such as the microsporidium *Nosema apis* ^18^, and systemic insecticides, such as neonicotinoids^56^ and fipronil^20^, might also affect drone survival or fertility.

**Figure 1:**
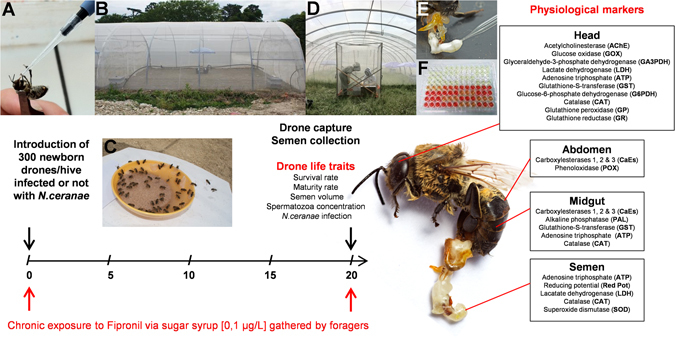
Experimental design. Drones were exposed to the pathogen *N. ceranae*, the insecticide fipronil or both under semi-field conditions. Newborn drones were individually fed a sugar syrup solution contaminated or not with 50,000 spores of *N. ceranae* (**A**) and cloistered in queenless colonies placed under a tunnel covered with an insect-proof net (**B**). For 20 days, colonies were supplied daily by foragers that gathered in a feeder with contaminated sugar syrup solution with fipronil at 0.1 µg.L^−1^ (**C**). At the end of the experiment, drones were caught (**D**) to collect semen in a glass capillary (**E**). Following the exposures, drone life traits were investigated, and analyses of physiological markers in the head, abdomen, midgut and semen were performed (**F**).

To study the effects of pathogen/pesticide co-exposure, we exposed drones in semi-field conditions from emergence to sexual maturity to the microsporidium *Nosema ceranae*, which is suspected to be a major cause of honey bee decline in Southern Europe^57^, or the insecticide fipronil, which is known to impair drone fertility^20, 58^, (Fig. 1). We sought to determine whether the combination of (i) exposure to an environmental concentration of 0.1 µg.L^−1^ fipronil^59^ in food brought to the hive and (ii) contamination with the 50,000 spores of the parasite *Nosema ceranae* could induce synergistic effects in male honey bees, as previously reported in workers^37, 38^. In our study, the effects on drones were assessed with an original approach examining drone fertility, life traits and primary physiological functions (neural, metabolic, immune, detoxification and antioxidant functions) using a battery of physiological markers in different biological compartments (see Materials and Methods section for more details, Fig. 1 and Table 1). These physiological markers were chosen for their relevance to assess the effects of anthropogenic pollutants^43, 60–62^, biological agents^36, 63^, and nutritional deficiency^63^ on honey bee physiology and were quantified in the head, abdomen, midgut, and semen. We then discuss the implication of these results on honey bee reproduction and their consequences on queen impoverishment, which leads to queen failure and is suspected to be a major cause of colony loss in winter^64–66^.

**Table 1:** Effects of the stressors, alone or combined, on physiological markers in drones.

Physiological marker	Tissue	unit	Physiological functions	Physiological markers responses (Mean ± SD)							
				Control		*Nosema ceranae*		Fipronil [0.1 µg.L^−1^]		Nos + Fip [0.1 µg.L^−1^]	
AChE	head	mUA.min^−1^.mg of tissue^−1^	neural	253.9 ± 30.2	**a**	235.9 ± 35.0	**ab**	221.2 ± 27.0	**b ↘**	226.7 ± 15.8	b ↘
GOX	head	mUA.min^−1^.mg of tissue^−1^	immune	32.3 ± 14.6	**a**	32.1 ± 13.5	**a**	34.0 ± 18.7	**a**	30.5 ± 18.7	a
GA3PDH	head	mUA.min^−1^.mg of tissue^−1^	metabolic	325.1 ± 54.3	**a**	334.5 ± 41.1	**ab**	336.7 ± 39.6	**ab**	391.8 ± 41.1	b ↗
LDH	head	mUA.min^−1^.mg of tissue^−1^	metabolic	46.5 ± 4.0	**a**	47.0 ± 3.8	**a**	52.5 ± 5.2	**b ↗**	53.0 ± 3.4	b ↗
ATP	head	LI.mg of tissue^−1^	metabolic	11117.6 ± 1023.4	**a**	11779.7 ± 900.8	**ab**	11171.3 ± 587.7	**a**	12421.8 ± 823.2	b ↗
GST	head	mUA.min^−1^.mg of tissue^−1^	metabolic. detoxification. antioxidant	252.3 ± 26.5	**a**	253.2 ± 28.2	**a**	232.8 ± 23.0	**ab**	228.3 ± 15.2	b ↘
G6PDH	head	mUA.min^−1^.mg of tissue^−1^	metabolic. antioxidant	7.8 ± 0.7	**a**	7.1 ± 1.3	**a**	7.2 ± 1.0	**a**	7.1 ± 0.9	a
CAT	head	mUA.min^−1^.mg of tissue^−1^	antioxidant	139.5 ± 51.9	**a**	164.3 ± 58.4	**ab**	158.1 ± 99.0	**ab**	244.9 ± 54.7	b ↗
GP	head	mUA.min^−1^.mg of tissue^−1^	antioxidant	24.9 ± 4.4	**a**	25.0 ± 4.4	**a**	31.01 ± 6.31	**b ↗**	32.8 ± 5.2	b ↗
GR	head	mUA.min^−1^.mg of tissue^−1^	antioxidant	20.4 ± 5.6	**a**	21.6 ± 4.4	**a**	23.1 ± 3.9	**ab**	26.0 ± 4.9	b ↗
CaE-1	abdomen	mUA.min^−1^.mg of tissue^−1^	metabolic. detoxification	316.6 ± 66.4	**a**	275.5 ± 39.4	**a**	265.8 ± 32.8	**a**	294.4 ± 54.4	a
CaE-2	abdomen	mUA.min^−1^.mg of tissue^−1^	metabolic. detoxification	518.9 ± 48.3	**a**	523.8 ± 29.4	**a**	501.8 ± 32.6	**a**	506.2 ± 24.5	a
CaE-3	abdomen	mUA.min^−1^.mg of tissue^−1^	metabolic. detoxification	70.1 ± 14.8	**a**	66.9 ± 8.1	**a**	68.7 ± 9.5	**a**	72.6 ± 15.2	a
POX	abdomen	mUA.min^−1^.mg of tissue^−1^	immune	2.1 ± 0.8	**a**	2.5 ± 1.0	**a**	3.3 ± 0.7	**b ↗**	3.3 ± 0.6	b↗
CaE-1	midgut	mUA.min^−1^.mg of tissue^−1^	metabolic. detoxification	910.7 ± 172.3	**a**	913.2 ± 134.0	**a**	1003.0 ± 140.7	**a**	993.8 ± 115.5	a
CaE-2	midgut	mUA.min^−1^.mg of tissue^−1^	metabolic. detoxification	536.5 ± 94.3	**ab**	521.5 ± 119.2	**a**	607.1 ± 61.8	**b**	577.8 ± 66.7	ab
CaE-3	midgut	mUA.min^−1^.mg of tissue^−1^	metabolic. detoxification	209.2 ± 49.3	**a**	211.6 ± 42.1	**a**	260.5 ± 45.2	**b↗**	280.8 ± 37.4	b ↗
ALP	midgut	mUA.min^−1^.mg of tissue^−1^	immune. metabolic	19.4 ± 5.6	**ac**	14.0 ± 4.7	**b ↘**	20.5 ± 6.4	**c**	16.0 ± 5.5	ab
ATP	midgut	LI.mg of tissue^−1^	metabolic	23798.2 ± 7663.7	**a**	28557.6 ± 12897.7	**a**	22123.8 ± 9941.7	**a**	22613.4 ± 9276.1	a
GST	midgut	mUA.min^−1^.mg of tissue^−1^	metabolic. detoxification. antioxidant	203.3 ± 39.7	**a**	204.4 ± 37.1	**a**	222.0 ± 25.0	**a**	254.3 ± 27.7	b ↗
CAT	midgut	mUA.min^−1^.mg of tissue^−1^	antioxidant	1154.8 ± 315.6	**a**	1414.8 ± 208.4	**bc ↗**	1345.4 ± 330.8	**ab**	1606.4 ± 313.8	c ↗

A multiple markers approach was performed to study the effects of the stressors in the head, midgut and abdomen of drones. Each marker was associated with one or more of the main physiological functions (neural, metabolic, immune, detoxification and antioxidant). The data represent the mean values ± standard deviations of the physiological markers responses expressed in milliUnits of Absorbance (mUA) or in Luminescence Intensity (LI) for ATP. Three samples of 6 drones were measured from each hive (n = 12 for each modality of treatment). For statistical analyses, a generalized linear mixed model was applied considering a random effect of the hives from which the drones came. Significant differences at *P* ≤ 0.05 between modalities are expressed with different letters. Case with the same letters indicate no significant difference between the groups. Arrows indicate the direction of the modulation relative to the control group.

## Results

### Drone life history traits

**Figure 2:**
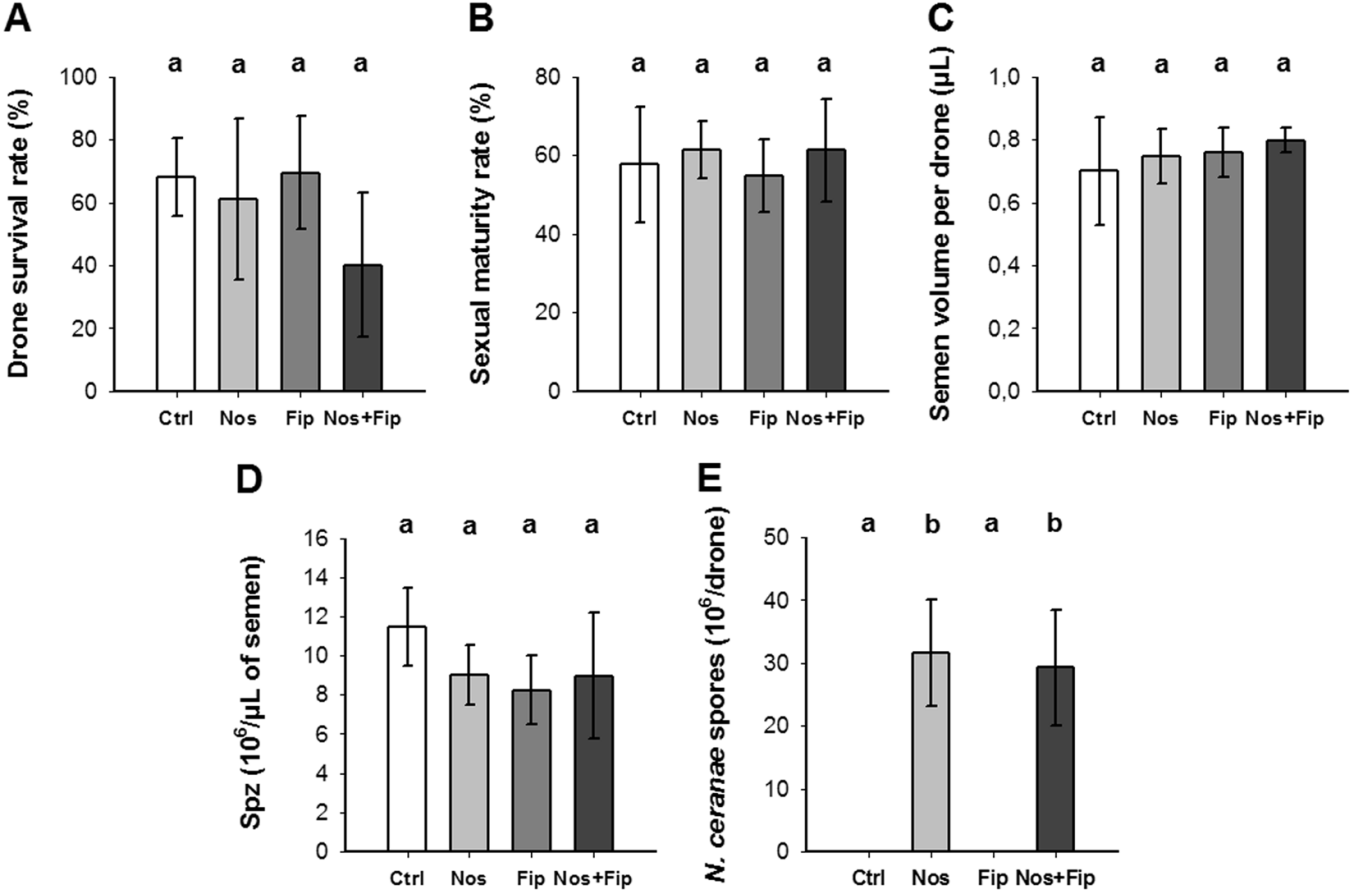
Effects of the stressors, alone or combined, on drone life traits. The effects of the pathogen and the insecticide alone or in combination were studied at the individual level. The survival rate (**A**), the sexual maturity rate (**B**), the semen volume per drone (**C**) and the spermatozoa concentration (**D**) were measured for each hive (n = 4 for each stressor modality). The level of *N. cerana*e infection (**E**) was determined using 3 samples of 5 drones per hive (n = 12 for each modality of treatment). The data represent the mean values ± standard deviations. Statistical analyses were performed using a post-hoc Wilcoxon test for the first four parameters (**A**–**D**). A generalized linear mixed model was applied to the level of *N. cerana*e infection (**E**) considering a random effect of the hives from which the drones came. Bars with different letters indicate a significant difference at (or below) the 0.05 level between groups.

The survival and sexual maturity rates of drones, the semen volume per drone, the spermatozoa concentration in semen and the level of *N. ceranae* contamination were recorded at the end of a 20-day exposure period. For survival and maturity rates, semen volume and spermatozoa concentration, no significant differences were observed (n = 4 per modality, Fig. 2), although in some cases, trends seemed to emerge. For the *N. ceran*ae/fipronil interaction, the survival rate dropped from approximately 70% to 40%, suggesting a synergistic effect (Fig. 2A). Moreover, although maturity rate and semen volume were not affected (Fig. 2B and C), the spermatozoa concentration decreased from the concentration of 11.5 × 10^6^ spermatozoa.µL^−1^ observed in controls to 9, 8.2 and 9 × 10^6^ spermatozoa.μL^−1^ in the *N. ceranae*, fipronil and *N. ceranae*/fipronil combination groups, respectively (Fig. 2D), corresponding to a decrease of more than 20%. Among all studied parameters, the only significant difference was found for the parasite load in the intestine between the infected bees and the other groups. The infection level of 20-day-old drones with *N. ceranae* (n = 12 per modality) revealed a parasite load of approximately 30 × 10^6^ spores per individual for the *N. ceranae* and *N. ceranae*/fipronil stressors (*P* < 0.001 in both cases, Fig. 2E). The control and fipronil groups exhibited a parasite load of approximately 40,000 spores. Furthermore, no *N. ceranae* spores were observed in drone sperm (data not shown), in contrast to the sperm contamination observed in drones of a similar age exposed to *N. apis* ^18^.

### Physiological markers in individual drones

Various physiological markers were tested in three biological compartments (head, midgut, and abdomen). These markers are linked to one or more key physiological functions related to the immune system (glucose oxidase (GOX), phenoloxidase (POX) and alkaline phosphatase (ALP)), the xenobiotic detoxification system (glutathione-S-transferase (GST) and carboxylesterases 1, 2 & 3 (CaEs)), antioxidant defense (GST, glucose-6-phosphate dehydrogenase (G6PDH), catalase (CAT), glutathione peroxidase (GP), glutathione reductase (GR)), neural activity (acetylcholinesterase (AChE)) or metabolism (ALP, G6PDH, glyceraldehyde-3-phosphate dehydrogenase (GA3PDH), lactate dehydrogenase (LDH), adenosine triphosphate (ATP), CaE-1, CaE-2, CaE-3, GST) (Table 1).

In drones infected with *N. ceranae*, significant effects were observed only in the midgut for ALP (*P* = 0.018) and CAT (*P* = 0.025) (Table 1); ALP was decreased, whereas CAT was increased. These results suggest changes to immune defenses, antioxidant activity, and metabolism. After sublethal chronic exposure to fipronil, significant effects on the drones were observed in all compartments and on five physiological functions studied. AChE was negatively regulated (*P* = 0.017), whereas LDH, GP, POX and CaE-3 increased (*P* = 0.012, *P* = 0.04, *P* = 0.007 and *P* = 0.004, respectively). Finally, the changes observed from each stressor alone were also observed for the *N. ceranae*/fipronil combination except for ALP. In the midgut, compared with each stressor alone, the changes in CAT and CaE-3 were enhanced by the combination of stressors, which appeared to generate specific effects in comparison with the controls (*P* = 0.0012 and *P* ≤ 0.001, respectively). Moreover, it appears that the combination of the stressors exerted specific effects that were significantly different from what was observed in the controls (GA3PDH (*P* = 0.038), ATP (*P* = 0.017), GST in head (*P* = 0.045), GST in midgut (*P* < 0.0001), CAT in head (*P* = 0.049), and GR (*P* = 0.01)). However, with the exception of GST in the midgut, for which the combination induced a synergistic effect, for the other parameters, the combination of the stressors did not elicit effects different from those induced in the head by either *N. ceranae* (GA3PDH, ATP and CAT) or fipronil (GA3PDH, GST, CAT and GR). Interestingly, GST was increased in the midgut and decreased in the head, indicating tissue-dependent regulation of the synergetic effect.

**Figure 3:**
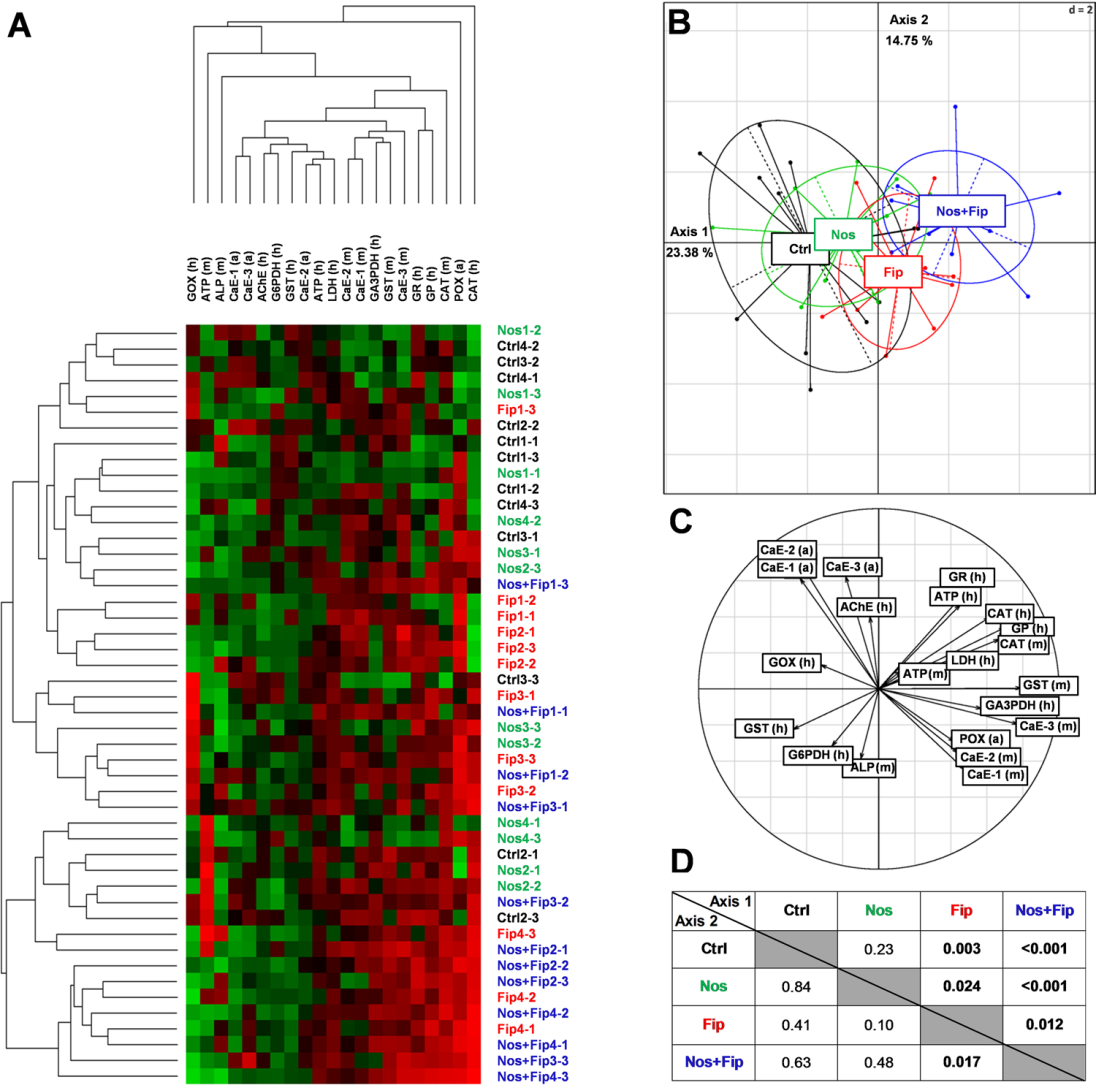
Effects of the stressors, alone or combined, on the physiological state of drones. According to the stressor, cluster analysis (hierarchical clustering results) after optimal reorganization^100^ (**A**) and principal component analysis (**B**) show a global approach of changes to the physiological markers in the head (h), midgut (m) and abdomen (a) of drones. A color code is assigned to each treatment: “black” for the control group (Ctrl), “green” for the *N. ceranae* group (Nos), “red” for the fipronil group (Fip), and “blue” for the *N. ceranae*/fipronil group (Nos + Fip). In the cluster analysis, the first number indicates the hive, and the second number indicates the sample. The intensity of modulation in the clusters is illustrated by the range of colors, with green and red indicating that a physiological marker was downregulated or upregulated, respectively, in comparison with the mean value in the controls. Black indicates no change in comparison with the mean value in the controls. The correlation circle (**C**) indicates the significance of the markers in the PCA representation. For statistical analysis, a post-hoc Wilcoxon test was applied to the values of the two main axes of the PCA. *P* values are indicated in the table (**D**).

Cluster analysis of the physiological markers showed a tendency of replicates to group according to the stressor administered to the honey bee drones (Fig. 3A). The clusters are more homogeneous for controls and the stressor combination (*N. ceranae*/fipronil). Principal component analysis (PCA) provides a visual representation of the physiological states of drones exposed to different stressors (Fig. 3B–D) and discriminated all of the treatments (Fig. 3B). Moreover, the *N. ceranae*/fipronil stressor was clearly distinct from the control. In the head and midgut, physiological makers linked to metabolism, antioxidant defenses and detoxification systems appeared to have the largest influence distinguishing the physiological state of drones according to the stressor experienced (Fig. 3C). The statistical analyses performed on the values of the two main axes of the PCA showed a significant difference between fipronil and the control and between *N. ceranae*/fipronil and all of the other treatments (Fig. 3D).

### Physiological markers for fertility in drone sperm

**Figure 4:**
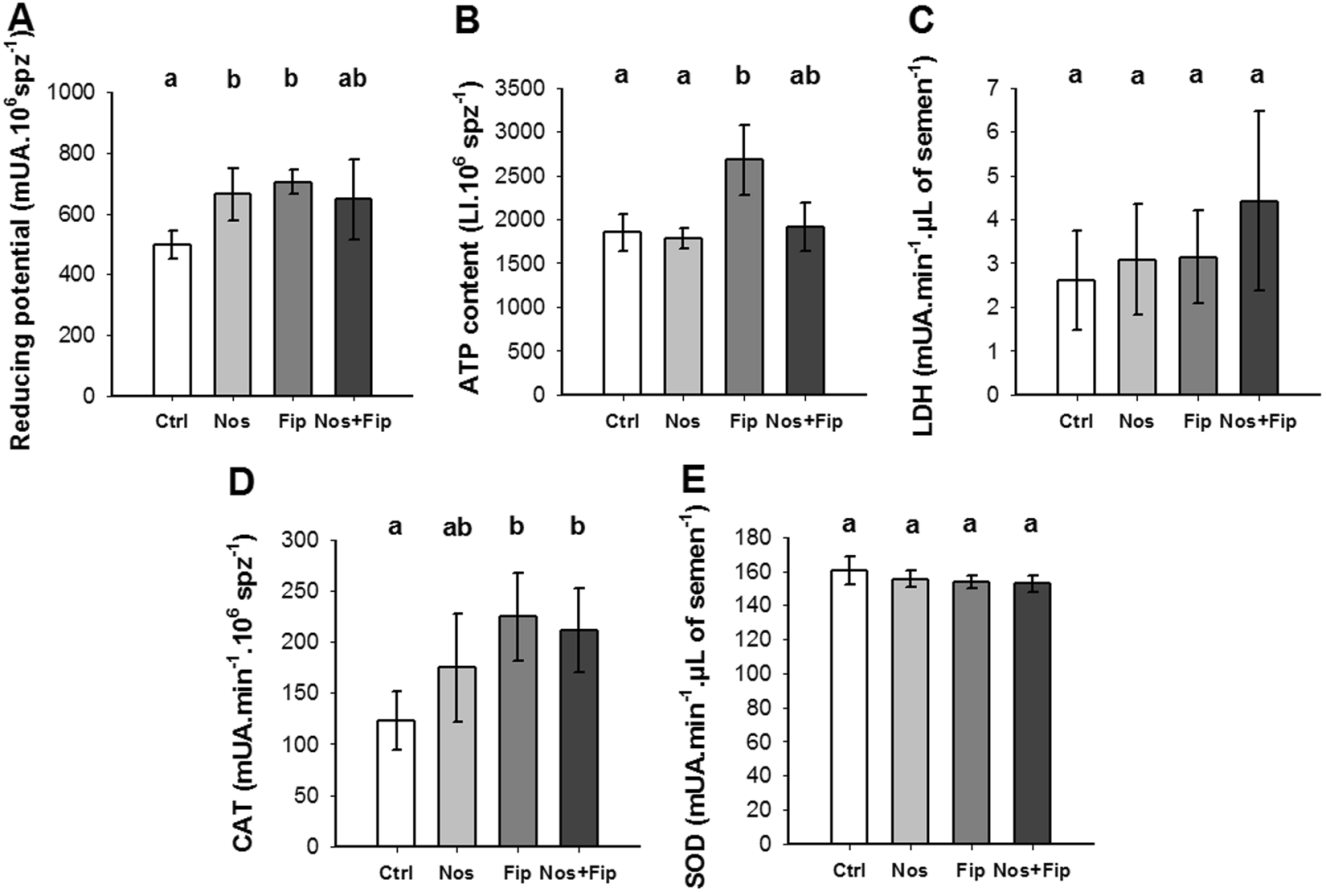
Effects of the stressors, alone or combined, on physiological markers in semen. Physiological markers were studied in semen. The reducing potential (**A**) and ATP content (**B**) in semen and the LDH activity in seminal fluid (**C**) were measured to assess the effects of each treatment on sperm metabolism. The CAT activity in spermatozoa (spz) (**D**) and the SOD activity in seminal fluid (**E**) were measured to assess the antioxidant response. The data represent the mean values ± standard deviations of the parameters expressed in milliUnits of Absorbance (mUA) for Red Pot, LDH, CAT and SOD, or in Luminescence Intensity (LI) for ATP (n = 4 for each modality of treatment). Statistical analyses were performed using a post-hoc Wilcoxon test. Significant differences at *P* ≤ 0.05 between modalities are expressed with different letters.

Physiological markers were studied in semen. Each marker is linked to one key physiological function (CAT, superoxide dismutase (SOD) for antioxidant ability and LDH, ATP and reducing potential (Red Pot) for metabolism) (Fig. 4, for more details see Materials and Methods section). In response to *N. ceranae* infection, significant effects were observed only in the Red Pot of drone sperm (Fig. 4A, *P* ≤ 0.05), although a trend toward positive CAT modulation was observed in the spermatozoa (Fig. 4D) in comparison with the control. After sublethal chronic exposure to fipronil, significant positive changes were observed in sperm for Red Pot and ATP values as well as for CAT in spermatozoa (*P* ≤ 0.05 in both cases) in comparison with the control. Finally, for the *N. ceranae/*fipronil interaction, a significant positive change was observed only for CAT in spermatozoa (Fig. 4D, *P* ≤ 0.05) in comparison with the control. However, positive tendencies for Red Pot (Fig. 4A) and LDH (Fig. 4C) were observed in seminal fluid. Interestingly, no change in spermatozoa ATP levels was detected (Fig. 4B). In all cases, it was notable that the effects induced by the combination of stressors were not significantly different from those induced by each of the stressors independently. In seminal fluid, no SOD changes (Fig. 4E) were observed, in contrast to CAT activity in spermatozoa (Fig. 4D), suggesting a compartment-dependent response to oxidative stress.

### Integrative analysis of all physiological markers

**Figure 5:**
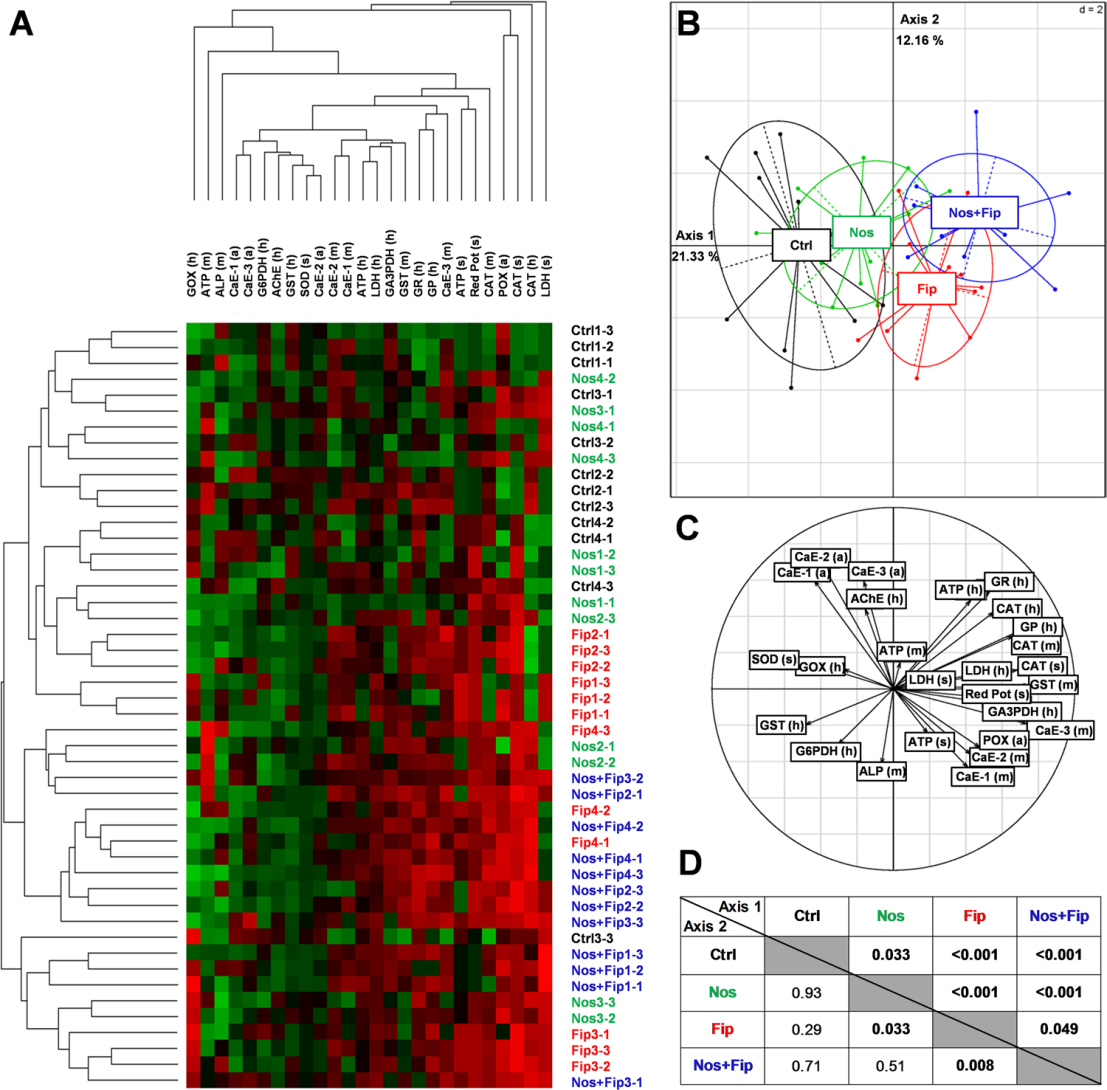
Effects of the stressors, alone or combined, on the physiological state of drones including semen. The cluster analysis (hierarchical clustering results) after optimal reorganization^100^ (**A**) and the principal component analysis (**B**) show an integrative approach to assess the physiological markers studied in the head (h), midgut (m), abdomen (a) and semen (s) of drones. A color code is assigned to each treatment: “black” for the control group (Ctrl), “green” for the *N. ceranae* group (Nos), “red” for the fipronil group (Fip), and “blue” for the *N. ceranae*/fipronil group (Nos + Fip). In the cluster analysis, the first number indicates the hive, and the second number indicates the sample. The intensity of modulation in the clusters is illustrated by the range of colors, with green and red indicating that a physiological marker was downregulated or upregulated, respectively, in comparison with the mean value in the controls. Black indicates no change in comparison with the mean value in the controls. The correlation circle (C) indicates the significance of the markers in the PCA representation. For statistical analysis, a post-hoc Wilcoxon test was applied on the values of the two main axes of the PCA. *P* values are indicated in the table (**D**).

In further cluster and PCA analyses (Fig. 5), we integrated the fertility parameters observed in sperm, seminal fluid and spermatozoa with the previous analyses involving only the head, midgut, and abdomen compartments (Fig. 3). Thus, all of the physiological markers and fertility parameters were used to compare the effects of different treatment modalities. Samples from three out of four stressor groups (*i.e*., Control, fipronil, *N. ceranae*/fipronil) were grouped. Interestingly, *N. ceranae* samples maintained heterogeneous distribution in the total cluster, which has been previously observed (Fig. 5A). Additionally, integrative PCA clearly discriminated between all of the stressors with significant differences between all of the groups (Fig. 5B–D). Interestingly, physiological parameters measured in semen and those measured in the head and midgut linked to metabolism, antioxidant defenses and detoxification systems were the key physiological markers explaining the separation of the experimental groups (Fig. 5C).

## Discussion

In ecosystems of the biosphere, each environmental stressor has the ability to disturb organisms from the molecular to population level. The effects can range from small cellular changes to the death of individuals and might include behavioral and reproductive disorders. However, in ecosystems, organisms are seldom exposed to only one abiotic or biotic stressor and are often exposed simultaneously to several environmental stressors. In combination, these stressors can elicit different types of effects, including additive, synergistic and antagonistic effects^30, 34^. Based on previous studies of honey bee workers exhibiting a synergistic effect from the pathogen/insecticide association *N. ceranae*/fipronil, we hypothesized that a synergetic effect might also occur in reproductive castes, particularly drones, which have a critical role in the life cycle of a honey bee colony. Indeed, drones have a key role ensuring genetic diversity via polyandry and also control semen quality, which the queen’s reproductive success depends on^20, 49–51^. Thus, survival, mating success and semen quality are key criteria whose integrity must be preserved in honey bee colonies. To this end, individuals possess a cellular arsenal (immune, detoxification, and antioxidant systems) to respond to biotic and abiotic stress factors or combinations of these factors. Thus, effects of the *N. ceranae*/fipronil combination on the drones were investigated from the physiological to the phenotypic level following the exposure of drones, in a semi-controlled environment, from emergence to sexual maturity.

According to the effects on the life history traits and physiological parameters at the individual scale, without considering semen, differential action of the stressors, which was moderate and limited to the midgut for *N. ceranae* and very important and widespread for the *N. ceranae*/fipronil (Table 1, Figs 2 and 3), was observed. Exposure to *N. ceranae* induced a strongly localized response in the midgut by interfering with immunity, metabolism and defenses against oxidative stress (Table 1). These localized disturbances might be explained by the fact that *N. ceranae* is a honey bee parasite specific to intestinal cells that disrupts the homeostasis of the digestive system^57^ by hijacking ATP in the host midgut epithelium^67^ and by affecting its defense mechanisms^40, 57, 68^. However, although males showed disturbances in defense systems, physiological markers revealed no effects on energetic metabolism, particularly regarding ATP levels. In any case, the survival of drones (Fig. 2A) was not engaged, as has been observed in honey bee strains tolerant to *N. ceranae* ^69^ but not in strains susceptible to *N. apis* ^18^ and *N. ceranae* ^69^. Moreover, the heterogeneity of drone response to the microsporidia, as observed in cluster analysis (Fig. 3A), potentially explaining the presence of individuals exhibiting different levels of pathogen tolerance, which might explain the moderate global impact of infection on the overall physiological state of drones (Fig. 3B–D). Regarding chronic exposure to insecticide, all of the studied physiological functions (metabolism, nervous system, immune system, detoxification system and defenses against oxidative stress) were disturbed in all of the biological compartments. However, fipronil and *N. ceranae* do not impact the same physiological markers, which is revealed as specificity in the mode of action of the two stressors (Table 1). In the honey bee, fipronil is known to disturb cellular metabolism^20, 43, 70^, the nervous system^71, 72^, the detoxification system^43, 61^ and immunity^40, 72^ and to elicit changes in the antioxidant system^61^ that are linked to the production of reactive oxygen species, as in other organisms^73^. The general effect of the insecticide on drones reveals systemic properties in the honey bee, similar to those observed in other animals^74^ and in plants^75^. However, despite the general effect, survival does not appear to be affected at this level of foraged food contamination as recently observed by Kairo *et al*.^20, 58^. Nevertheless, when considering (*i*) previous studies showing the ability of fipronil to alter the cognitive functions of workers^76–80^, (*ii*) AChE changed in drones (*i.e*., the enzyme involved in the cholinergic synapses) (Table 1) and (*iii*) the weakening of individuals, revealed by the generalized physiological disturbances (Table 1 and Fig. 3B–D), it is legitimate to think that exposure to fipronil might alter a drone’s ability to compete with non-exposed congeners in the drone congregation area and to mate with the queen. Upon co-exposure (*N. ceranae*/fipronil), the effects induced by *N. ceranae* and fipronil alone were still observed and tended to be increased in some cases. In addition, 6 new markers were affected, confirming the specific effects of the interaction in comparison with the controls, which could lead to a significant synergetic effect, as observed for midgut GST (Table 1 and Fig. 3). Thus, the overall physiological state of individuals exposed to the combined stressors was different from that of individuals in the other groups and was completely distinct from that of the control individuals (Fig. 3B–D). These results might explain the excess drone mortality upon co-exposure to *N. ceranae* and fipronil that was clearly observed in honey bee workers^37, 38^. Under the co-exposure conditions of this study, the adverse impact was not limited to a failure in mating success, as mentioned above for fipronil, but in cases in which survival is committed, they might be extended to a drone population drop in congregation areas. This drop in drone population could result in a lower selective pressure and, in turn, in the mating of drones that are less vigorous and less adapted to their habitats. This scenario would reinforce the assumptions highlighting a decrease of healthy drones to explain the lower quality of queens observed in apiaries^55, 81, 82^. Thus, even if fertility aspects are not considered, impairments of male integrity could lead to poor queen quality and, consequently, could be sufficient to alter the development of “daughter colonies”.

Regarding fertility, the results suggest that semen quality is impacted regardless of stressor. These changes result in metabolic disturbances and affect oxidative stress defense, which were both physiological functions studied in sperm (Fig. 4). Interestingly, the physiological damage induced by both stressors at the individual level was also observed in the sperm. Moreover, each stressor had a significant impact on fertility parameters whereas at the individual level, differential effects occur. This finding was supported by an integrative analysis of all of the measured physiological markers (individuals including semen) showing that all of the groups can be clearly distinguished (Fig. 5). The fact that males exposed to *N. ceranae* exhibit a different physiological state from all of the other groups (control, fipronil and the combination of *N. ceranae*/fipronil) suggests that reproductive functions are more sensitive to stressors than other studied functions. Regardless of stressor, fertility impairment is observed at the phenotypic level, with a decline in sperm concentration of approximately 20%, whereas the maturity and semen volume of the drones are not affected (Fig. 2B–D). Although these results are not statistically significant and only a trend for an effect has emerged, in the case of fipronil, the results are highly consistent with those of a previous study clearly showing the effects of insecticide on both sperm concentration and metabolism with similar disorders^20, 58^. Thus, whatever the nature of the stress factor considered in this study at the individual level, semen quality appears altered in the same way. Hence, even if queens were not affected by a shortage of drones able to mate, they would likely be affected by the poor quality of the semen received. This finding suggests that these effects influence not only the reproductive success of a queen, as has been observed with males exposed to fipronil, but also the offspring^20^.

In conclusion, this study is the first in the available literature to show specific effects of an insecticide/pathogen combination (*N. ceranae*/fipronil) on the males of a eusocial insect species. Similar effects have been shown on the survival and physiology of drones but not on their fertility. Indeed, the results have shown effects on sperm quality regardless of stress factor applied (*N. ceranae* and fipronil alone or in combination). Consequently, the high sensitivity of fertility parameters suggests that reproduction could be one of the first functions of drones affected in stressful conditions. As a result, stresses exerted alone might not affect the vitality and survival of individuals but could have many effects on reproductive functions. All of the changes observed in the drones or their fertility could have serious consequences for the life cycle of an *A. mellifera* colony. Even if the physical integrity of the drone was preserved, allowing him normal behavior and unchanged mating success, damage would result by transmitting poor quality semen, affecting not only queen performance but also the offspring. If the physiology of drones is strongly impacted to jeopardize their survival, mating performance might be compromised and lead to a shortage of healthy males in congregation areas. This might lead to poorly mated queens related to decreased selection pressure or a potential loss of genetic diversity, which would be largely detrimental to the species. This study highlights the need to study the effects of stress factor combinations on fertility problems in the honey bee as one of the mechanistic explanations for queen failure. This study also highlights the need for a multi stressor approaches when studying reproductive disorders affecting many species in the biosphere.

## Materials and Methods

### Experimental design

Drones were exposed to the insecticide fipronil and/or the pathogen *N. ceranae* under semi-field conditions in Avignon (South France) between early June and late July of 2012. To perform this experiment and control drone age, 15 queens were previously encaged within 15 hives on drone combs for laying. Twenty-four days after laying, newly emerging drones from these 15 rearing hives were homogenously mixed before being introduced to the experimental hives. Thus, the control and treated hives contained drones of same age and genetic diversity. The drones were then reared from emergence to sexual maturity for 20 days in sixteen queenless colonies, which are known to take better care of drones^83^. These queenless colonies were placed in compartmented tunnels (two hives/compartment), covered with an insect-proof net to control the foraging of food. Homogeneous batches of 300 drones were cloistered in these queenless colonies containing 5000 workers, 1 brood comb and 5 empty combs for food storage, according to a previously described protocol^20, 84^. Drones, worker bees and brood combs needed for the study were obtained from honey bee colonies that were monitored for their sanitary status and specially checked for the absence *Nosema* spp. infection.

#### N. ceranae experimental infection and exposure to fipronil

To test the effects of *N. ceranae*/fipronil interaction on drones, 4 experimental groups of 4 queenless colonies each were established: the control group (Ctrl), a group infected with *N. ceranae* (Nos), a group exposed to fipronil (Fip) and a group both infected with *N. ceranae* and exposed to fipronil (Nos + Fip). Before introduction to queenless colonies, emerging drones were individually fed 2 µL sugar syrup (50%, w/v) with or without 50,000 spores of *N. ceranae*, which is a realistic infectious load^85^ (Fig. 1A). Fresh spores were isolated from the midguts of forager bees from local colonies that were naturally infected with the microsporidium^36^. To confirm that the species *N. ceranae* was used in the experiments, distinction between *N. apis* and *N. ceranae* was performed via standard PCR^86^. Under the tunnel, colonies were daily supplied by foragers that collected sugar syrup, crushed pollen and water in feeders outside the colony (Fig. 1B and C). The sugar syrup (50%, w/v, 0.1% DMSO), with or without fipronil at the relevant environmental concentration of 0.1 µg.L^−1^ (fipronil is likely to be found in nectar and pollen at this concentration^59^) was provided daily from 8:30 a.m. to 11:30 a.m. Crushed pollen and water were available the rest of the time^20^. Thus, drones were chronically exposed to the insecticide in the hive via food gathered by foragers mimicking natural exposure conditions. Twenty days after introduction, the surviving drones were caught (Fig. 1D), and the endophallus was manually everted to collect semen in a glass capillary using an insemination syringe^87^ (Fig. 1E). Fresh drone semen samples from the same colony were pooled in a glass capillary and kept at 21 °C in the dark until analysis the next day. Drone bodies were frozen immediately at −80 °C after semen collection for further analysis. Thus, the effects induced by *N. ceranae*, fipronil and the combination of both stressors were investigated from the drone life traits to the main physiological functions using physiological markers linked to neural, metabolic, detoxification, antioxidant and immune functions (Fig. 1, Table 1).

In this study, it was difficult to perform a large number of measurements because (*i*) the protocol required the individual feeding of 4800 newborn drones to expose them to the microsporidium, (*ii*) fertility and physiological parameters required a large volume of semen, and a mature drone produces less than 1 µL, and (*iii*) some parameters, such as survival rate, can be measured only at the colony level.

#### Drone life traits

After 20 days of exposure in the hive, drone life traits such as survival, maturity, infection with *N. ceranae*, semen volume and spermatozoa concentration were investigated. The drone survival rate was estimated by counting the remaining surviving drones. Because we considered the possibility that stressors could modify the maturity rate of drones by acting as endocrine disruptors, this parameter was assessed by measuring the number of drones able to provide sperm after stimulation. Then, the overall semen volume per colony was determined during the semen collection process. The average semen volume per drone was calculated from the two previous parameters. The semen concentration was estimated by counting spermatozoa under a phase contrast microscope using a Neubauer improved/Petroff counting cell. Semen was diluted (1:1500) in Kiev solution (36 g/L trisodium citrate, 3.6 g/L sodium bicarbonate, 0.6 g/L potassium chloride, 5 g/L glucose, 3 g/L sulfanilamide, pH 8.5, osmotic pressure 460 mOs/mL)^20^ and counted in triplicate. Considering that spermatogenesis has been initiated at time of drone emergence and that the number of sperm is fixed before the exposure period considered in this study, the spermatozoa concentration is assessed as a parameter for evaluating the cytotoxicity of stressors in sperm^58^. For each of these previous parameters, one measure per colony was obtained and four colonies were investigated (n = 4 for each modality). To determine the level *N. ceranae* infection in 20 day-old drones, 3 samples of 5 drones per hive were analyzed (n = 12 for each modality). Briefly, abdomens were crushed in distilled water, and the resulting suspension was filtered. The spore concentration of the suspension was determined using a Malassez counting cell^36^.

#### Analysis of physiological markers

To study the effects of different stressors on drone physiology, enzymatic and non-enzymatic markers were measured in the head, midgut, abdomen and semen (Fig. 1, Table 1). All of the examined enzymes and metabolites were chosen because they are representative of the targeted physiological functions, i.e., detoxification, the neural, antioxidant and immunity systems and metabolism (Table 1). Analyses were conducted in the head, abdomen, intestine and sperm because while the response of the organism to stress can be localized at the point of infection or entry of the xenobiotic, such as the intestine, there might also be systemic repercussions in another parts of the organism. Thus, the activities of AChE, GOX, GA3PDH, G6PDH, GP and GR were measured in the head. Activities of POX, ALP and SOD were measured in the abdomen devoid of midgut, midgut and spermatozoa, respectively. The ATP content was measured in the head, midgut and semen, and the Red Pot was also measured in semen. GST activity was measured in the head and midgut. LDH activity was measured in the head and diluted seminal fluid. CAT activity was measured in the head, midgut and spermatozoa, and CaEs were measured in the midgut and abdomen (Fig. 1 and Table 1). In the head, midgut and abdomen, 3 samples of 6 drones each were analyzed per hive (n = 12 for each modality). For semen, one measure per colony was performed (n = 4 for each modality). For each sample, each biological parameter was determined in triplicate with an infinite^®^ F500 plate reader from TECAN (Lyon, France) (Fig. 1F).

#### Sample preparation

For assays in the head, midgut and abdomen, the tissue from 6 drones was homogenized in lysis buffer [10 mM NaCl, 1% (w/v) Triton X-100, protease inhibitors (2 µg.mL^−1^ antipain, leupeptin and pepstatin A, 25 units.mL^−1^ aprotinin and 0.1 mg.mL^−1^ soybean trypsin inhibitor), 40 mM sodium phosphate, pH 7.4] to obtain a 10% (w/v) extract^88^. Tissues were homogenized with a Tissue-Lyser II (Qiagen^®^) homogenizer for 5 periods of 10 s at 30 Hz separated by an interval of 30 s. After 10 min, the homogenization procedure was repeated a second time. The homogenate was centrifuged for 20 min at 15000 g, and the supernatant was used to analyze enzymes activities and non-enzymatic compounds in the biological compartments. For enzymatic assays in semen, the semen was diluted in an equal volume of Kiev solution and centrifuged for 20 min at 16000 g to separate spermatozoa and seminal fluid^89^. The supernatant, corresponding to seminal fluid, was recovered to measure LDH and SOD activities. The pellet, corresponding to spermatozoa, was rinsed twice by suspension in Kiev solution, centrifuged and recovered. Then, the pellet was diluted 10% (v/v) in lysis buffer to lyse the spermatozoa. The cellular debris were removed by centrifugation for 15 min at 15000 g before measuring CAT activity. All of the procedures were performed at 4 °C. Unlike enzymatic activity measurements, the ATP content and the reducing potential were measured in non-separated semen at room temperature.

#### Acetylcholinesterase (AChE)

AChE is a neural enzyme involved the controls nerve transmission by hydrolyzing the neurotransmitter acetylcholine in the synapse. Head AChE activity was determined, spectrophotometrically at 412 nm in reaction medium containing 0.3 mM acetylthiocholine iodide (AcSCh.I), 1.5 mM 5,5′-dithiobis-2-nitrobenzoic acid (DTNB) and 100 mM sodium phosphate (Na_3_PO_4_) pH 7.0 according to the method of Ellman *et al*.^90^ as modified by Belzunces *et al*.^88^.

#### Glucose oxidase (GOX)

GOX catalyzes the oxidation of D-glucose into D-gluconic acid and hydrogen peroxide (H_2_O_2_), which has antimicrobial properties. Excreted in honey by salivary glands, this enzyme is involved in the social immunity of honey bees. Head GOX was assayed by assessing the oxidation of *o-*dianisidine by H_2_O_2_ at 430 nm. The reaction medium contained 100 mM glucose, 2.5 Units/200 µL peroxidase, 0.3 mM *o*-dianisidine and 125 mM potassium phosphate pH 7.0^36^.

#### Phenol oxidase (POX)

POX plays a role in the individual immunity of insects through the melanization process involved in tissue regeneration and encapsulation of foreign bodies, such as pathogens. Abdomen POX activity was determined by following the conversion of 3,4-dihydroxy-L-dihydroxyphenylalanine (L-DOPA) into melanin at 490 nm. The reaction medium contained 20 mM NaCl, 0.4 mg.mL^−1^ L-DOPA and 10 mM sodium phosphate (NaH_2_PO_4_) pH 7.2^36^.

#### Alkaline phosphatase (ALP)

ALP plays an important role maintaining the homeostasis of gut tissues due to its involvement in many metabolic processes linked to immune responses. The midgut PAL dephosphorylating activity was monitored by the conversion of p-nitrophenyl phosphate p-NPP) into p-nitrophenol at 410 nm. The reaction medium contained 20 µM magnesium chloride (MgCl_2_), 2 mM p-NPP and 100 mM Tris-HCl pH 8.5^91^.

#### Carboxylesterases 1, 2 & 3 (CaEs)

CaEs are involved in numerous metabolic and detoxification processes. Midgut and abdomen CaE-1, CaE-2 and CaE-3 were assayed according to their respective specific substrate α-naphthyl acetate (α-NA), β-naphthyl acetate (β-NA) or p-nitrophenyl acetate (p-NPA), respectively. The reaction medium contained 0.01 mM acetylcholinesterase inhibitor BW284C51, 0.1 mM α-NA, β-NA or p-NPA and 100 mM sodium phosphate (NaH2PO4) pH 7.0. For CaE-1 and CaE-2, catalysis proceeded for 1 min and was stopped by adding 0.2 reaction volume of a solution containing 10% sodium dodecyl sulfate (SDS) and 4 mg.mL^−1^ fast garnet GBC sulfate salt. The absorbance was read at 568 nm and 515 nm, respectively. CaE-3 activity was continuously monitored at 410 nm^92^.

#### Adenosine triphosphate (ATP)

ATP, the energy fuel of the cell, is also a coenzyme involved in numerous metabolic reactions. ATP content in the head, midgut and semen was determined using ATPlite kit (PerkinElmer) based on luminescence measurements produced by the oxidation of D-luciferin by luciferase that involves 1 molecule of ATP and O_2_^84, 93^.

#### Reducing potential (Red Pot)

The Red Pot corresponds to the ability of cells to reduce compounds linked to metabolic activity. The Red Pot in semen was assessed using a Prestoblue kit (Invitrogen). The assay was based on the reduction of the cell permeable compound resazurin to a red fluorescent resorufin. The absorbance of resorufin was measured at 570 nm^84^.

#### Glyceraldehyde-3-phosphate dehydrogenase (GA3PDH)

GA3PDH reversibly catalyzes the conversion of glyceraldehyde-3-phosphate (GA3P) in the presence of nicotinamide adenine dinucleotide (NAD^+^) into 1,3-biphosphoglyceric acid (1,3-BPG) and the reduced form of nicotinamide adenine dinucleotide (NADH) during glycolysis and plays an important role in energetic metabolism. GA3PDH can also catalyze the reverse reaction when gluconeogenesis overtakes glycolysis. GA3PDH activity was determined in the head with the reverse reaction using 3-phosphoglyceric acid (3-PGA), which is converted by phosphoglycerate kinase (PGK) into 1,3-BPG. 1,3-BPG is converted to GA3P in the presence of NADH whose oxidation was followed at 340 nm. The reaction medium contained 7 mM 3-PGA, 4 mM L-cysteine-HCL neutralized with sodium bicarbonate (NaHCO_3_), 2 mM magnesium sulfate (MgSO_4_), 120 µM NADH, 1.2 mM ATP, 1 mM ethylenediaminetetraacetic acid (EDTA), 5 units.mL^−1^ 3-phosphoglycerate kinase (3-PGK), 80 mM triethanolamine buffer pH 7.0^43^.

#### Glucose-6-phosphate dehydrogenase (G6PDH)

G6PDH is a key enzyme of the pentose phosphate pathway that generates NADPH, which contributes to the regeneration of reduced glutathione (GSH) involved in the defenses against oxidative stress. This metabolic pathway also contributes to the biosynthesis of nucleotides, amino acids and some fatty acids involved in cell metabolism. G6PDH catalyzes the conversion of glucose-6-phosphate (G-6-P) in the presence of the oxidized form of nicotinamide adenine dinucleotide phosphate (NADP^+^) into 6-phosphogluconolactone and the reduced form of nicotinamide adenine dinucleotide phosphate (NADPH). Head G6PDH activity was determined by continuously following the formation of NADPH at 340 nm. The reaction medium contained 100 mM Tris-HCl buffer at pH 7.4, 10 mM MgCl_2_, 1 mM G-6-P, 0.5 mM NADP^+^ and 100 mM Tris-HCl pH 7.4^43^.

#### Lactate dehydrogenase (LDH)

LDH catalyzes the conversion of pyruvate into lactate in the presence of NADH. In anaerobic conditions, LDH enables the regeneration of NAD^+^ used in the glycolysis pathway. The regeneration of NAD^+^ was followed at 340 nm to determine LDH activity in the head and seminal fluid. The reaction medium contained 5 mM EDTA, 0.2 mM NADH, 2 mM sodium pyruvate and 50 mM triethanolamine pH 7.6^94^.

#### Glutathione-S-transferase (GST)

GST is mainly involved in the reaction conjugating GSH to exogenous compounds such as xenobiotics or endogens products from cell metabolism such as reactive oxygen species (ROS). Thus, GST plays a role in xenobiotic detoxification, antioxidant defense and metabolic regulation. GST activity in the head and midgut was determined by measuring the conjugation of GSH to 1-chloro-2,4-dinitrobenzene (CDNB) at 340 nm. The reaction medium contained 1 mM EDTA, 2.5 mM GSH, 1 mM CDNB and 100 mM Na/K phosphate pH 7.4^95^.

#### Superoxide dismutase (SOD)

SOD converts the superoxide anion (O_2_^.−^) into hydrogen peroxide (H_2_O_2_) to limit oxidative stress. SOD activity was indirectly measured in seminal fluid using the xanthine/xanthine oxidase system to generate O_2_^.−^ and nitro blue tetrazolium (NBT). SOD competes with xanthine oxidase and limits the generation of reduced NBT, which was followed at 560 nm. The reaction medium contained 0.1 mM EDTA, 0.1 mM xanthine, 0.025 mM NBT, 0.00833 U/mL xanthine oxidase, and 50 mM phosphate/carbonate pH 7.8^84^.

#### Glutathione peroxidase (GP)

The GP catalyzes the destruction of peroxides, such as H_2_O_2_, by oxidizing GSH and generating H_2_O and oxidized glutathione (GSSG). Thus, GP contributes to the regulation of reactive oxygen species (ROS) involved in oxidative stress. Head GP was assayed using tert-butyl hydroperoxide (TBHP) as the substrate. The generated GSSG was reduced by glutathione reductase (GR) in the presence of NADPH to generate GSH and NADP. The conversion of NADPH in NADP^+^ was followed at 340 nm. The reaction medium contained 1 mM EDTA, 0.2 mM TBHP, 0.85 mM GSSG, 0.16 mM NADPH, 0.25 U/mL GR and 50 mM Na/K phosphate pH 7.4^68^.

#### Glutathione reductase (GR)

As described above, GR enables the regeneration of GSH involved in the regulation of oxidative stress. Head GR was followed at 340 nm by the conversion of NADPH into NADP^+^. The reaction medium contained 1 mM EDTA, 0.85 mM GSSG, 0.16 mM NADPH and 50 mM Na/K phosphate pH 7.4^68^.

#### Catalase (CAT)

The CAT catalyzes the decomposition of H_2_O_2_ into oxygen (O_2_) and water (H_2_O) to protect cells against oxidative stress. The decomposition of H_2_O_2_ by CAT was followed in head, midgut and spermatozoa at 240 nm. The reaction medium contained 30 mM H_2_O_2_ and 100 mM sodium phosphate pH 7.0^96^.

### Statistical Analysis

Parameters for which one measure per hive were performed (*i.e*., drone survival rate, sexual maturity rate, semen volume per drone, spermatozoa concentration in semen and physiological markers in semen; n = 4 per modality of treatment) used a post-hoc Wilcoxon test because the small number of data points required a non-parametric test. Parameters for which several measures per hive were performed (*i.e*., physiological markers response in drones and the level of *N. ceranae* infestation; n = 12 per modality of treatment) used a generalized linear mixed model with random effect on the hive from which drones came. These statistical analyses were performed using the package “lme4” in R software^97^. Bars with the same letters indicate no significant difference between the groups. Bars with different letters indicate a significant difference at the 0.05 level between groups.

The effects of different treatments on drone physiology were described with 2 complementary approaches. A principal component analysis (PCA) was performed using the package “ade4” in the R software^98^. To compare groups from the PCA representation, a post-hoc Wilcoxon test was applied on the values of the two main axes of the PCA. In addition, a hierarchical classification of data was performed using PermutMatrix software (adaptation of the Eisen method) for analyzing and visualizing data^99^. The distance measure used was Euclidian distance, with UPGM as the linkage rule for clusters. For the latter approach, data normalization was required to convert each measure to the rate of variation compared with the average of controls. These analyses were performed considering physiological marker responses, first in drone and then in drones plus semen.

## References

[1] Pellati D (2008). Genital tract infections and infertility. Eur. J. Obstet. Gynecol. Reprod. Biol..

[2] Moretti E, Federico MG, Giannerini V, Collodel G (2008). Sperm ultrastructure and meiotic segregation in a group of patients with chronic hepatitis B and C. Andrologia.

[3] Feki NC (2009). Semen quality decline among men in infertile relationships: experience over 12 years in the south of tunisia. J. Androl..

[4] Mnif W (2011). Effect of endocrine disruptor pesticides: a review. Int. J. Environ. Res. Public Health.

[5] Multigner L, Oliva A (2001). Environment as a risk factor for male infertility. Sci. World J..

[6] Levine H, Swan SH (2015). Is dietary pesticide exposure related to semen quality? Positive evidence from men attending a fertility clinic. Hum. Reprod..

[7] Tyler CR, Jobling S, Sumpter JP (1998). Endocrine disruption in wildlife: a critical review of the evidence. Crit. Rev. Toxicol..

[8] Hutchinson TH (2002). Reproductive and developmental effects of endocrine disrupters in invertebrates: *in vitro* and *in vivo* approaches. Toxicol. Lett..

[9] Kohler HR, Triebskorn R (2013). Wildlife ecotoxicology of pesticides: can we track effects to the population level and beyond?. Science.

[10] Bauer LS, Nordin GL (1989). Effect of nosema-fumiferanae (microsporida) on fecundity, fertility, and progeny performance of choristoneura-fumiferana (lepidoptera, tortricidae). Environ. Entomol..

[11] Del Cacho E, Marti JI, Josa A, Quilez J, Sanchez Acedo C (1996). Effect of Varroa jacobsoni parasitization in the glycoprotein expression on Apis mellifera spermatozoa. Apidologie.

[12] Duay P (2002). Relation between the level of preimaginal infestation by the broodmite Varroa destructor and adult life expectancy in drone honeybees (Hymenoptera: Apidae: Apis mellifera). Entomol. Gen..

[13] Seth RK, Kaur JJ, Rao DK, Reynolds SE (2004). Effects of larval exposure to sublethal concentrations of the ecdysteroid agonists RH-5849 and tebufenozide (RH-5992) on male reproductive physiology in Spodoptera litura. J. Insect Physiol..

[14] Gauthier L (2011). Viruses associated with ovarian degeneration in Apis mellifera L. queens. PLoS One.

[15] Brennan LJ, Haukedal JA, Earle JC, Keddie B, Harris HL (2012). Disruption of redox homeostasis leads to oxidative DNA damage in spermatocytes of Wolbachia-infected Drosophila simulans. Insect. Mol. Biol..

[16] Collins AM, Pettis JS (2013). Correlation of queen size and spermathecal contents and effects of miticide exposure during development. Apidologie.

[17] Misra S (2014). Identification of Drosophila-based endpoints for the assessment and understanding of xenobiotic-mediated male reproductive adversities. Toxicol. Sci..

[18] Peng Y, Baer-Imhoof B, Millar AH, Baer B (2015). Consequences of Nosema apis infection for male honey bees and their fertility. Sci. Rep..

[19] Williams GR (2015). Neonicotinoid pesticides severely affect honey bee queens. Sci. Rep..

[20] Kairo G (2016). Drone exposure to the systemic insecticide Fipronil indirectly impairs queen reproductive potential. Sci. Rep..

[21] Chaimanee V, Evans JD, Chen YP, Jackson C, Pettis JS (2016). Sperm viability and gene expression in honey bee queens (Apis mellifera) following exposure to the neonicotinoid insecticide imidacloprid and the organophosphate acaricide coumaphos. J. Insect Physiol..

[22] McCallum ML (2013). Endocrine disruption of sexual selection by an estrogenic herbicide in the mealworm beetle (Tenebrio molitor). Ecotoxicology.

[23] Knight AL, Flexner L (2007). Disruption of mating in codling moth (Lepidoptera: Tortricidae) by chlorantranilipole, an anthranilic diamide insecticide. Pest Manag. Sci..

[24] Laycock I, Lenthall KM, Barratt AT, Cresswell JE (2012). Effects of imidacloprid, a neonicotinoid pesticide, on reproduction in worker bumble bees (Bombus terrestris). Ecotoxicology.

[25] Tassou KT, Schulz R (2013). Low field-relevant tebufenozide concentrations affect reproduction in Chironomus riparius (Diptera: Chironomidae) in a long-term toxicity test. Environ. Sci. Pollut. Res. Int..

[26] Sandrock C (2014). Sublethal neonicotinoid insecticide exposure reduces solitary bee reproductive success. Agric. For. Entomol..

[27] Costa MA (2014). Sublethal and transgenerational effects of insecticides in developing Trichogramma galloi (Hymenoptera: Trichogrammatidae). Ecotoxicology.

[28] Xiao D, Yang T, Desneux N, Han P, Gao X (2015). Assessment of sublethal and transgenerational effects of pirimicarb on the wheat aphids Rhopalosiphum padi and Sitobion avenae. PLoS One.

[29] Pigeault R, Vezilier J, Nicot A, Gandon S, Rivero A (2015). Transgenerational effect of infection in Plasmodium-infected mosquitoes. Biol. Lett..

[30] Sures B (2004). Environmental parasitology: relevancy of parasites in monitoring environmental pollution. Trends Parasitol..

[31] Vidau C (2011). Fipronil is a powerful uncoupler of oxidative phosphorylation that triggers apoptosis in human neuronal cell line SHSY5Y. Neurotoxicology.

[32] Potts SG (2010). Global pollinator declines: trends, impacts and drivers. Trends. Ecol. Evol..

[33] Vanbergen AJ, the Insect Pollinators Initiative (2013). A-Threats to an ecosystem service: pressures on pollinators. Front. Ecol. Environ..

[34] Holmstrup M (2010). Interactions between effects of environmental chemicals and natural stressors: a review. Sci. Total Environ..

[35] Relyea R, Hoverman J (2006). Assessing the ecology in ecotoxicology: a review and synthesis in freshwater systems. Ecol. Lett..

[36] Alaux C (2010). Interactions between Nosema microspores and a neonicotinoid weaken honeybees (Apis mellifera). Environ. Microbiol..

[37] Vidau C (2011). Exposure to sublethal doses of fipronil and thiacloprid highly increases mortality of honeybees previously infected by Nosema ceranae. PLoS One.

[38] Aufauvre J (2012). Parasite-insecticide interactions: a case study of Nosema ceranae and fipronil synergy on honeybee. Sci. Rep..

[39] Retschnig G, Neumann P, Williams GR (2014). Thiacloprid–Nosema ceranae interactions in honey bees: host survivorship but not parasite reproduction is dependent on pesticide dose. J. Invert. Pathol..

[40] Aufauvre J (2014). Transcriptome analyses of the honeybee response to Nosema ceranae and insecticides. PLoS One.

[41] Sanchez-Bayo F (2016). Are bee diseases linked to pesticides? - A brief review. Environ. Int..

[42] Dussaubat C (2016). Combined neonicotinoid pesticide and parasite stress alter honeybee queens’ physiology and survival. Sci. Rep..

[43] Renzi MT (2016). Chronic toxicity and physiological changes induced in the honey bee by the exposure to fipronil and Bacillus thuringiensis spores alone or combined. Ecotoxicol. Environ. Saf..

[44] Goulson D, Nicholls E, Botias C, Rotheray EL (2015). Bee declines driven by combined stress from parasites, pesticides, and lack of flowers. Science.

[45] Neumann P, Carreck N (2010). Honey bee colony losses. J. Apic. Res..

[46] van Engelsdorp D, Meixner MD (2010). A historical review of managed honey bee populations in Europe and the United States and the factors that may affect them. J. Invertebr. Pathol..

[47] Winston, M. L. *The biology of the honeybee* (Harvard University Press, 1987).

[48] Baer B (2005). Sexual selection in Apis bees. Apidologie.

[49] Tarpy DR (2003). Genetic diversity within honeybee colonies prevents severe infections and promotes colony growth. Proc. R. Soc. B Biol. Sci..

[50] Mattila HR, Seeley TD (2007). Genetic diversity in honey bee colonies enhances productivity and fitness. Science.

[51] Oldroyd BP, Fewell JH (2008). Large fitness benefits from polyandry in the honey bee, Apis mellifera. Trends. Ecol. Evol..

[52] Rinderer TE, De Guzman LI, Lancaster VA, Delatte GT, Stelzer JA (1999). Varroa in the mating yard: I. The effects of Varroa jacobsoni and Apistan (R) on drone honey bees. Am. Bee J..

[53] Sylvester HA, Watts RP, De Guzman LI, Stelzer JA, Rinderer TE (1999). Varroa in the mating yard: II. The effects of Varroa and fluvalinate on drone mating competitiveness. Am. Bee J..

[54] Collins AM, Pettis JS (2001). Effect of varroa infestation on semen quality. Am. Bee J..

[55] Burley LM, Fell RD, Saacke RG (2008). Survival of honey bee (Hymenoptera: Apidae) spermatozoa incubated at room temperature from drones exposed to miticides. J. Econ. Entomol..

[56] Straub L (2016). Neonicotinoid insecticides can serve as inadvertent insect contraceptives. Proc. R. Soc. Lond. B Biol. Sci..

[57] Higes M, Martin-Hernandez R, Meana A (2010). Nosema ceranae in Europe: an emergent type C nosemosis. Apidologie.

[58] Kairo, G. *et al*. Assessment of the toxicity of pesticides on honey bee drone fertility using laboratory and semi-field approaches: a case study of fipronil. *Environ. Toxicol. Chem*. doi:10.1002/etc.3773 (2017). 10.1002/etc.3773 28224659

[59] Bonmatin JM (2015). Environmental fate and exposure; neonicotinoids and fipronil. Environ. Sci. Pollut. Res. Int.

[60] Badiou-Beneteau A (2013). Honeybee biomarkers as promising tools to monitor environmental quality. Environ. Int..

[61] Carvalho SM, Belzunces LP, Carvalho GA, Brunet JL, Badiou-Beneteau A (2013). Enzymatic biomarkers as tools to assess environmental quality: a case study of exposure of the honeybee Apis mellifera to insecticides. Environ. Toxicol. Chem..

[62] Wegener J (2016). Secondary biomarkers of insecticide-induced stress of honey bee colonies and their relevance for overwintering strength. Ecotoxicol. Environ. Saf..

[63] Di Pasquale G (2013). Influence of pollen nutrition on honey bee health: do pollen quality and diversity matter?. PLoS One.

[64] vanEngelsdorp D, Hayes J, Underwood RM, Pettis J (2008). A survey of honey bee colony losses in the US, fall 2007 to spring 2008. PLoS One.

[65] Brodschneider R, Moosbeckhofer R, Crailsheim K (2010). Surveys as a tool to record winter losses of honey bee colonies: a two year case study in Austria and South Tyrol. J. Apic. Res..

[66] Genersch E (2010). The German bee monitoring project: a long term study to understand periodically high winter losses of honey bee colonies. Apidologie.

[67] Williams BA (2009). Unique physiology of host-parasite interactions in microsporidia infections. Cell. Microbiol..

[68] Dussaubat C (2012). Gut pathology and responses to the microsporidium Nosema ceranae in the honey bee Apis mellifera. PLoS One.

[69] Huang Q, Kryger P, Le Conte Y, Moritz RF (2012). Survival and immune response of drones of a Nosemosis tolerant honey bee strain towards N. ceranae infections. J. Invert. Pathol..

[70] Nicodemo D (2014). Fipronil and Imidacloprid reduce honeybee mitochondrial activity. Environ. Toxicol. Chem..

[71] Roat TC (2013). Effects of sublethal dose of fipronil on neuron metabolic activity of Africanized honeybees. Arch. Environ. Contam. Toxicol..

[72] Roat TC (2014). Modification of the brain proteome of Africanized honeybees (Apis mellifera) exposed to a sub-lethal doses of the insecticide fipronil. Ecotoxicology.

[73] Wang X (2016). Fipronil insecticide toxicology: oxidative stress and metabolism. Crit. Rev. Toxicol..

[74] Gibbons D, Morrissey C, Mineau P (2015). A review of the direct and indirect effects of neonicotinoids and fipronil on vertebrate wildlife. Environ. Sci. Pollut. Res. Int..

[75] Simon-Delso N (2015). Systemic insecticides (neonicotinoids and fipronil): trends, uses, mode of action and metabolites. Environ. Sci. Pollut. Res. Int..

[76] Decourtye A (2005). Comparative sublethal toxicity of nine pesticides on olfactory learning performances of the honeybee Apis mellifera. Arch. Environ. Contam. Toxicol..

[77] El Hassani AK, Dacher M, Gauthier M, Armengaud C (2005). Effects of sublethal doses of fipronil on the behavior of the honeybee (Apis mellifera). Pharmacol. Biochem. Behav..

[78] Aliouane Y (2009). Subchronic exposure of honeybees to sublethal doses of pesticides: effects on behavior. Environ. Toxicol. Chem..

[79] Bernadou A, Demares F, Couret-Fauvel T, Sandoz JC, Gauthier M (2009). Effect of fipronil on side-specific antennal tactile learning in the honeybee. J. Insect Physiol..

[80] Decourtye A (2010). Sublethal effects of fipronil on the ability of honeybees (Apis mellifera L.) to orientate in a complex maze. Julius-Kühn-Archiv..

[81] Camazine S (1998). How healthy are commercially-produced US honey bee queens?. Am. Bee J..

[82] Rhodes, J. & Somerville, D. Introduction and early performance of queen bees: some factors affecting success: a report for the Rural Industries Research and Development Corporation (Rural Industries Research and Development Corporation, 2003).

[83] Ruttner, F. The instrumental insemination of the queen bee (Apimondia, 1976).

[84] Ben Abdelkader F (2014). Semen quality of honey bee drones maintained from emergence to sexual maturity under laboratory, semi-field and field conditions. Apidologie.

[85] Meana A, Martín-Hernández R, Higes M (2010). The reliability of spore counts to diagnose Nosema ceranae infections in honey bees. J. Apic. Res..

[86] Higes M, Martín R, Meana A (2006). Nosema ceranae, a new microsporidian parasite in honeybees in Europe. J. Invert. Pathol..

[87] Cobey SW (2007). Comparison studies of instrumentally inseminated and naturally mated honey bee queens and factors affecting their performance. Apidologie.

[88] Belzunces LP, Lenoir-Rousseaux J-J, Bounias M (1988). Properties of acetylcholinesterase from Apis mellifera heads. Insect Biochem..

[89] Weirich GF, Collins AM, Williams VP (2002). Antioxidant enzymes in the honey bee, Apis mellifera. Apidologie.

[90] Ellman GL, Courtney KD, Andres V, Featherstone RM (1961). A new and rapid colorimetric determination of acetylcholinesterase activity. Biochem. Pharmacol..

[91] Bounias M, Kruk I, Nectoux M, Popeskovic D (1996). Toxicology of cupric salts on honeybees. V. Gluconate and sulfate action on gut alkaline and acid phosphatases. Ecotoxicol. Environ. Saf..

[92] Gomori G (1953). Human esterases. J. Lab. Clin. Med..

[93] Cree IA, Andreotti PE (1997). Measurement of cytotoxicity by ATP-based luminescence assay in primary cell cultures and cell lines. Toxicol. In Vitro.

[94] Al-Lawati H, Kamp G, Bienefeld K (2009). Characteristics of the spermathecal contents of old and young honeybee queens. J. Insect Physiol..

[95] Habig WH, Pabst MJ, Jakoby WB (1974). Glutathione S-transferases the first enzymatic step in mercapturic acid formation. J. Biol. Chem..

[96] Beers RF, Sizer IW (1952). A spectrophotometric method for measuring the breakdown of hydrogen peroxide by catalase. J. Biol. Chem..

[97] Bates D, Maechler M, Bolker B, Walker S (2015). Fitting linear mixed-effects models using lme4. J. Stat. Softw..

[98] Dray S, Dufour A-B (2007). The ade4 package: implementing the duality diagram for ecologists. J. Stat. Softw..

[99] Caraux G, Pinloche S (2005). PermutMatrix: a graphical environment to arrange gene expression profiles in optimal linear order. Bioinformatics.

[100] Bar-Joseph Z, Gifford DK, Jaakkola TS (2001). Fast optimal leaf ordering for hierarchical clustering. Bioinformatics.

